# Intrinsic Brain Abnormalities in Patients with Hepatitis C Virus Infection with Cognitive Impairment: A Preliminary Resting-State fMRI Study

**DOI:** 10.1155/2020/1693043

**Published:** 2020-11-03

**Authors:** Xiao-Hong Zhang, Jia-Yan Shi, Chuanyin Zhan, Ling Zhang, Hua-Jun Chen

**Affiliations:** ^1^Department of Radiology, Fujian Medical University Union Hospital, Fuzhou 350001, China; ^2^Department of Radiology, The First Affiliated Hospital of Nanjing Medical University, Nanjing 210029, China

## Abstract

**Purpose:**

Patients with a hepatitis C virus (HCV) infection frequently exhibit various neuropsychiatric complications such as cognitive decline. This study is aimed at investigating alterations in regional and network-level neural function in patients with HCV infection and examining the association between these alterations and patients' cognition dysfunction.

**Methods:**

The study included 17 patients with HCV infection and 17 healthy controls. These individuals had undergone resting-state functional magnetic resonance imaging as well as cognitive assessment using a battery of tests that were collectively called the “psychometric hepatic encephalopathy score (PHES)” examination. Analyses of amplitude of low-frequency fluctuation (ALFF) and seed-based functional connectivity (FC) were conducted to assess, respectively, regional neural function and functional integration.

**Results:**

HCV-infected patients performed significantly worse in cognitive tests. In the HCV group, ALFF decreased in Region 1 (left medial frontal gyrus and bilateral anterior cingulate gyrus) and Region 2 (right middle and superior frontal gyrus). The HCV group showed lower FC between Region 1 and right middle frontal gyrus, whereas they presented an increase in FC between Region 2 and the left supramarginal gyrus/superior temporal gyrus and right supramarginal gyrus. No significant correlation was observed between ALFF/FC measurements and PHES result.

**Conclusion:**

This preliminary study presents additional evidence that HCV infection affects brain function, including local intrinsic neural activity and global functional integration.

## 1. Introduction

There is increasing evidence that hepatitis C virus (HCV) infections not only cause hepatic symptoms but also directly affect the nervous system [1]. Recent studies have revealed the direct neuroinvasion of HCV based on the evidence of viral replication (reflected by replicative intermediate of HCV RNA and proteins) in the central nervous system [2]. Further evidence has demonstrated that human brain microvascular endothelial cells have functional receptors that support HCV tropism and replication, which in turn result in the apoptosis of these cells that consequently disrupt the blood-brain barrier [3]. Alterations in the blood-brain barrier then allow circulating inflammatory cytokines and chemokines to reach the brain, which subsequently contributes to the neurological disturbances [4].

In agreement with the evidences on HCV neuroinvasion, many HCV-infected patients (up to 50%) exhibit cognitive decline besides chronic fatigue and mood alterations [5]. There is increasing evidence of the negative impacts of HCV infection on cognitive functions such as attention and executive deficits, memory problems, and learning disability [6–9]. These cognitive declines in HCV-infected patients could severely affect an individual's social and physical activities, further influencing the quality of life of patients [4]. These cognitive dysfunctions are demonstrated to be associated with HCV infection-induced metabolic disorders (e.g., lower N-acetyl aspartate/creatine ratios involving the cerebral cortex and higher choline/creatine ratios in the basal ganglia and frontal white matter [8–10]), impairment of white matter microstructural integrity [11], and alterations in serotonergic and dopaminergic neurotransmission [12]. These patients significantly improve after successful eradication of HCV using antiviral therapy [6, 13].

Resting-state functional magnetic resonance imaging (fMRI) serves as a novel approach in measuring spontaneous brain activity by examining low-frequency fluctuations in the blood oxygenation level-dependent (BOLD) signal [14, 15]. It has been extensively utilized in various neuropsychiatric diseases, including Alzheimer's disease [16], Parkinson's disease [17], schizophrenia [18], and major depression [19], and has played a major role in unveiling the mechanism underlying these neurologic disorders [20, 21]. HCV infections can also induce alterations in spontaneous brain activity, given that the following indexes that reflect spontaneous neural function change in HCV-infected patients: (1) mean dominant frequency of electroencephalography (EEG) is slowed [9] and (2) the cerebral glucose metabolism is reduced in a few brain regions, including the limbic association cortex, frontal and parietal cortex, and superior temporal cortex [22]. Thus, resting-state fMRI may be potentially utilized in the assessment of brain functional alterations that are related to HCV infection.

In this exploratory study, we employ resting-state fMRI to assess regional and network-level brain functional changes in HCV patients. Analyses of the amplitude of low-frequency fluctuation (ALFF) and seed-based functional connectivity (FC) were performed to assess, respectively, regional neural function and functional integration. ALFF reflects regional spontaneous cerebral neural activity [16, 23], whereas FC reveals the functional coordination between various regions [21]. Correlation analyses were also conducted to investigate the association of these regional and network-level functional alterations with cognitive impairment in HCV patients. This preliminary investigation may provide new insight into the mechanisms underlying HCV-related effects on brain function.

## 2. Materials and Methods

### 2.1. Participants

This research study was approved by the local Research Ethics Committees. We obtained written informed consent from each subject before the study began. Seventeen HCV-infected patients recruited through the outpatient clinic and 17 healthy controls recruited from advertisements comprised this study. Demographic information of the participants is presented in Table 1. No significant differences in relation to age, gender, or education level were observed between the two groups. For each HCV-infected patient, the quantitative polymerase chain reaction (HCV-PCR) was performed to assess virus load (RNA). The average virus load (PCR titer) was (8.5 ± 3.7) × 10^6^ IU/mL (range : 0.95–1.43 × 10^6^ IU/mL) in the HCV-infected patient group. A part of HCV-infected (5 subjects) were undergoing treatment of interferon when they were recruited. A battery of cognitive tests called psychometric hepatic encephalopathy score (PHES) examination was used to assess cognition function in all participants. This examination included five subtests, namely, the number connection test A, number connection test B, digit symbol test, serial dotting test, and line tracing test. The cognitive test was performed as previously described [24].

The exclusion criteria used in this study were as follows: (1) a diagnosis of liver cirrhosis or severe fibrosis, (2) hepatic encephalopathy as well as other neuropsychiatric disorders, (3) currently being treated with psychotropic medications, and (4) a diagnosis of uncontrolled endocrine or metabolic disease (such as a thyroid dysfunction).

### 2.2. MRI Data Acquisition

MR imaging was conducted using a 3.0 T scanner (Siemens, Verio, Germany). Three-dimensional T1-weighted magnetization-prepared rapid gradient echo (MPRAGE) sagittal images were gathered with the following parameters: TR = 1.9 ms, TE = 2.48 ms, matrix = 256 × 256, flip angle = 9°, FOV = 256 mm × 256 mm, 176 slices, and slice thickness = 1.0 mm (without interslice gap). Functional images were obtained with an echoplanar imaging sequence with the following parameters: TR = 2,000 ms, TE = 25 ms, matrix = 64 × 64, flip angle = 90°, FOV = 240 mm × 240 mm, 35 contiguous axial slices, slice thickness = 4 mm (without interslice gap), and volume number = 180. The participants were asked to keep their eyes closed, refrain from thinking about anything specific, and remain still.

### 2.3. Functional MRI Data Preprocessing

The Statistical Parametric Mapping (SPM version 12, https://www.fil.ion.ucl.ac.uk/spm/) software and the Data Processing and Analysis for Brain Imaging (DPABI, http://rfmri.org/dpabi) tool were employed to preprocess the functional MRI data, as previous study [24, 25]. The first 10 volumes were excluded to decrease the influence of the unstable initial MR imaging signals. Afterwards, we performed slice timing correction and realignment for head motion correction. A subject was excluded when his/her translational movement was >2.0 mm or the rotation was >2.0° [26, 27]. The individual T1-weighted images were coregistered with the mean functional images. A unified segmentation algorithm was then employed to segment the transformed structural images into gray matter, white matter, and cerebrospinal fluid. Then, using the normalization parameters that were estimated during unified segmentation, the motion-corrected functional images were further normalized into the standard Montreal Neurological Institute (MNI) space and then resampled to 3 mm × 3 mm × 3 mm. Finally, the functional images were spatially smoothed with a Gaussian kernel at a 4 mm full width set at half maximum. The preprocessed functional MRI data were further linearly detrended and bandpass-filtered (0.01–0.08 Hz) to decrease low-frequency drift and high-frequency physiological respiratory and cardiac noise.

### 2.4. ALFF Analysis

The ALFF analysis referred to a previous study [16]. The time courses were translated to the frequency domain with a fast Fourier transform, and then, the square root of the power spectrum was estimated and averaged across 0.01–0.08 Hz at every voxel. This averaged square root was designated as the ALFF. Then, to decrease the global effects of variability among subjects, the ALFF in each voxel was divided by the mean ALFF value of the global gray matter of every subject, thereby generating a relative ALFF. The relative ALFF was then used in further statistical analyses.

One-sample *t*-tests were performed on the individual ALFF maps to assess the within-group ALFF pattern using a voxel-wise approach for each group. To explore the ALFF differences between two groups, two-sample *t*-tests were then performed. To correct for multiple comparisons at the cluster level, we performed family-wise error (FWE) correction (voxel *P* < 0.005 and cluster-level *P* < 0.05) on all statistical maps. The results were visualized using the BrainNet Viewer toolbox (http://www.nitrc.org/projects/bnv/) [28].

### 2.5. Functional Connectivity Analysis

The functional connectivity analysis was conducted, using the SPM software and DPABI toolbox. A seed-based temporal correlation approach was conducted for FC analysis. As shown below, ALFF changes in patients with HCV infection were observed in two prefrontal brain regions (Table 2), and these were chosen as seeds in the subsequent FC analyses. To regulate any possible influence of head motion as well as global white matter and cerebrospinal fluid signals on our findings, we employed linear regression to remove various sources of spurious variance such as six head motion parameters and mean signals of cerebrospinal fluid, white matter, and whole brain. After that, a reference time series for every seed region was obtained by averaging the resting-state fMRI time series of voxels of this seed region. Then, voxel by voxel Pearson correlation analysis was conducted between the reference time series and the time series of the rest of the brain. We then normalized the Pearson correlation maps with a Fisher *Z*-transform.

For each group, a one-sample *t*-test was employed to determine the FC spatial distribution pattern of every seed. The voxel-wise FCs were compared between the two groups, using a two-sample *t*-test. To correct for multiple comparisons at the cluster level, we conducted false discovery rate (FDR) correction (voxel *P* < 0.005 and cluster-level *P* < 0.05) on all statistical maps. The results were visualized using the BrainNet Viewer toolbox (http://www.nitrc.org/projects/bnv/) [28].

### 2.6. Correlation Analysis

The two areas with significant ALFF difference were designated as regions of interest (ROI). The ALFF values of these two ROIs were obtained. Then, we calculated the FC strengths between two ROIs and the regions with significant FC changes. Spearman correlation analysis was conducted to determine the correlation between these ALFF/FC values and the HCV-infected patients' cognitive performance. The FDR-corrected *P* < 0.05 were considered statistically significant.

## 3. Results

### 3.1. Cognitive Assessment Results

Compared with the HC group, HCV-infected patients performed significantly worse on most (4/5) cognitive tests, including the number connection test A, number connection test B, digit symbol test, and the line tracing test (Table 1). The PHES score was lower in the HCV group than that in the HC group. These results indicate significant cognitive deficits in HCV-infected patients.

### 3.2. Results of ALFF Analysis

**Figure 1:**
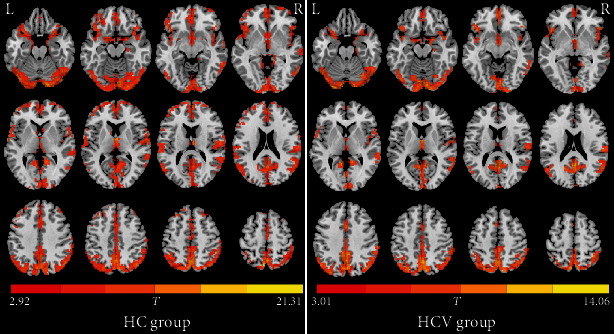
Within-group ALFF maps in the healthy control (HC) group as well as the patients infected with hepatitis C virus (HCV). The letters “L” and “R” represent the left and right sides, respectively.

**Figure 2:**
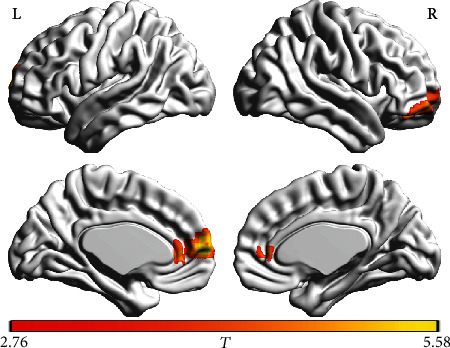
Brain regions showing decreased ALFF in patients with HCV infection. The patients showed lower ALFF values in two prefrontal regions, including ROI-1 (left medial frontal gyrus and bilateral anterior cingulate gyrus) and ROI-2 (right middle and superior frontal gyrus). The letters “L” and “R” represent the left and right sides, respectively.

The within-group ALFF maps are shown in Figure 1. By visual inspection, we noted that within the HC group, several brain regions such as the posterior cingulate gyrus and adjacent precuneus, medial prefrontal cortex and anterior cingulate gyrus, inferior parietal lobules, and occipital regions exhibited higher ALFF values relative to the other brain regions. This ALFF pattern well agreed with the results of previous reports [16, 18, 29, 30]. The ALFF pattern within the HCV group was highly similar to that in the HC group; however, the ALFF map in the HC group showed a broader pattern, relative to that in the HCV group. Statistically, the HCV group showed lower ALFF values in two prefrontal regions, including ROI-1 (left medial frontal gyrus and bilateral anterior cingulate gyrus) and ROI-2 (right middle and superior frontal gyrus) (Figure 2). These two regions are listed in Table 2.

### 3.3. Results of FC Analysis

**Figure 3:**
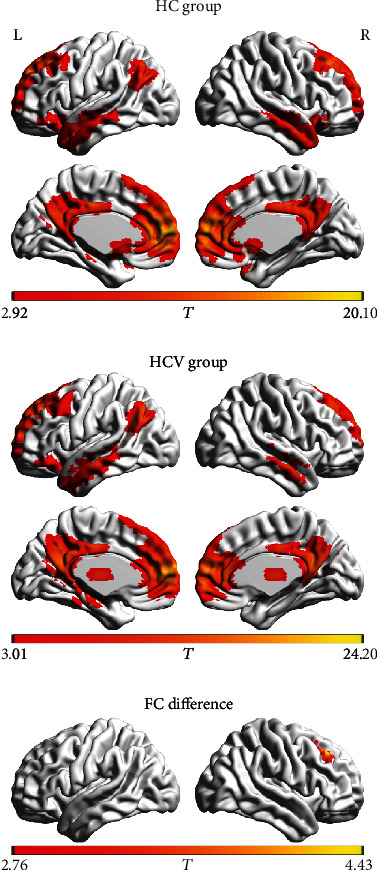
The functional connectivity pattern of seed region (i.e., left medial frontal gyrus and bilateral anterior cingulate gyrus) in the healthy control (HC group) and the patients with hepatitis C virus infection (HCV group). Compared with the HC group, the HCV group shows decreased functional connectivity between the seed region and right middle frontal gyrus. The letters “L” and “R” represent the left and right sides, respectively.

**Figure 4:**
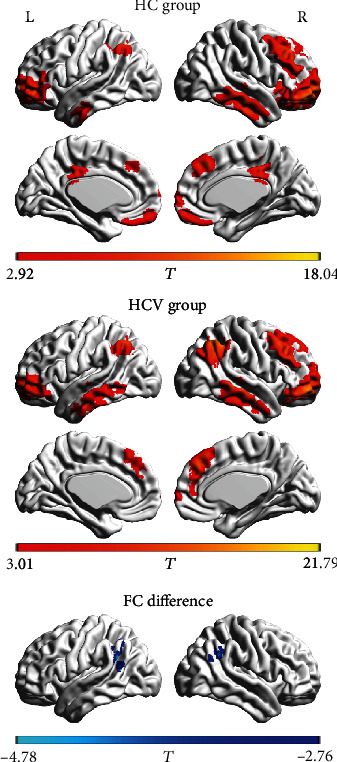
The functional connectivity pattern of seed region (i.e., right middle and superior frontal gyrus) in the healthy control (HC group) and the patients with hepatitis C virus infection (HCV group). Compared with the HC group, the HCV group shows increased functional connectivity between the seed region and the left supramarginal gyrus/superior temporal gyrus and the right supramarginal gyrus. The letters “L” and “R” represent the left and right sides, respectively.

Figures 3 and 4 show the results of further FC analysis. Compared with the HC group, the HCV group showed a decrease in FC between the ROI-1 and right middle frontal gyrus (Table 3). In contrast, the HCV group showed an increase in FC between the ROI-2 and the left supramarginal gyrus/superior temporal gyrus and the right supramarginal gyrus (Table 3).

### 3.4. Results of Correlation Analysis

No significant correlation was observed between ALFF/FC measurements and HCV-infected patients' cognitive performance reflected by PHES results, after FDR correction. This may be due to the small sample size that limited the statistical power, to some extent.

## 4. Discussion

To the best of our knowledge, the present study serves as one of the first investigations on alterations of brain spontaneous activity in HCV patients with cognitive impairment. The ALFF value, which is the index of regional neural activity, decreased in two prefrontal areas, i.e., left medial frontal gyrus and bilateral anterior cingulate gyrus and right middle and superior frontal gyrus. This finding agrees with the results of previous reports in which the abnormal neural activity related to HCV infection is demonstrated by EEG and PET (positron emission tomography) studies [9, 22]. Additionally, using these two frontal areas as seed regions, FC analyses showed a decrease in FC in the right middle frontal gyrus, as well as increased FC in the left supramarginal gyrus/superior temporal gyrus and right supramarginal gyrus. The results of FC analysis are in line with the findings of a previous report in which FC changes were identified in patients with HCV infection and correlate with cognitive alterations [31]. Thus, these findings may jointly point to a connectivity-based pathophysiologic process in HCV-infected patients with cognitive impairment.

The prefrontal cortex has been proven to be the pathological node frequently affected by HCV infection, in which the patients show a reduction in the neural energy metabolism and cerebral blood volume [22, 32]. Also, the trend of lower N-acetyl aspartate/creatine ratio has been noted in the anterior cingulate gyrus, which may suggest a decrease in neuronal activity in HCV-infected patients [32]. Given the above facts, it is expected that ALFF decreases in the frontal areas (i.e., left medial frontal gyrus/bilateral anterior cingulate gyrus and right middle and superior frontal gyrus) in HCV patients. The reduction in ALFF may imply the abnormality in regional neural activity and could induce the consequential defects in the relevant brain functions, as previous reports [16]. Given that several cognitive functions (such as attention/executive function and memory) are modulated by the prefrontal cortex [33–36], it is speculated that ALFF reduction may reflect the impairment in these cognitive functions in patients with HCV infection. In line with this speculation, the previous correlation analysis demonstrates the relationship between reduced cerebral glucose utilization in several prefrontal areas (e.g., medial frontal gyrus and anterior cingulate gyrus) and impaired cognitive functions (such as attention and memory deficits) in HCV-infected patients [22].

The FC pattern of the first seed region (left medial frontal gyrus and bilateral anterior cingulate gyrus) mainly covers the distribution of the default mode network. This network is demonstrated to be associated with various emotions (such as depression and anxiety) and cognition (such as attention and memory) processing, all of which are aberrant in HCV-infected patients [5, 7, 9]. Therefore, it is implied that functional dissociation in this network may contribute to the neural substrates of HCV-related neurocognitive and neuropsychiatric disorders. However, an increase in FC was observed between the second seed region (right middle and superior frontal gyrus) and bilateral parietal-temporal cortex. The FC increment could be considered a compensatory procedure in response to the neurological disruption due to HCV neuroinvasion [37]. This plausible mechanism of compensation may be in agreement with previous findings: (1) by magnetic resonance spectroscopy, the increased N-acetyl-aspartate plus N-acetyl-aspartyl-glutamate concentration is demonstrated in the case of HCV infection, which is considered a compensatory process to preserve neuronal function [38], and (2) by resting-state fMRI, the increased FC in right posterior parietal regions is identified, which has been hypothesized to reflect functional reorganization that is associated with a compensatory mechanism to supply additional attention resources for better memory performance in HCV patients [31].

This study has several limitations. First, the data are cross-sectional, and thus, future longitudinal studies are warranted to demonstrate the full-time course of functional alteration as well as its causal relationship to HCV infection. Second, this study had a relatively small sample size, which limited statistical power. Further investigation using a larger cohort is recommended to validate our results. Third, several patients with interferon treatment were included in this study. Interferon neurotoxicity in the treatment of viral hepatitis has been documented [39, 40]; therefore, whether the intrinsic brain abnormalities investigated in this study is attributed to the effect of interferon, to some extent, should be further clarified in the future. Fourth, this study did not assess mood alterations, which are another common neuropsychiatric symptom (manifested by higher levels of anxiety and depression [9]) that is accompanied by cognitive impairment in HCV-infected patients. Psychological symptoms such as anxiety and depression are able to cause abnormal changes in the intrinsic brain function [19, 41]. Thus, the absence of psychological status screening may hamper our interpretation of the results, to some extent. Fifth, only a simplified cognitive assessment (PHES examination) was applied in our study. More comprehensive tests are recommended in the future study to examine the abnormal cognitive status in HCV-infected patients, which is beneficial to identify the key characteristics of cognitive impairments and their relationships with regional and network-level brain dysfunction. Finally, we only performed two seed-based FC analyses to reflect network-level brain functional changes. More comprehensive exploration (e.g., whole-brain FC analysis) is recommended in the future.

In this preliminary study, we applied ALFF and FC analyses to reflect, respectively, regional spontaneous brain activity and functional coordination between various regions, which contributed to improving our understanding of the neural mechanism underlying the dysfunctions related to HCV infection. Our results revealed that HCV-infected patients had aberrant regional brain function and altered FC between distinct regions that are primarily associated with attention and executive dysfunction. Thus, our study provides further evidence that HCV infection affects brain function. Further investigation using a larger cohort should be conducted to determine the relationship between the intrinsic brain abnormalities and HCV infection-related cognitive dysfunctions.

## Figures and Tables

**Table 1:** Demographic and clinical information of the study participants.

	HC subjects (*n* = 17)	Patients with HCV infection (*n* = 17)	*P* value
Age (year)	46.0 ± 13.0	47.1 ± 13.1	0.822
Gender (male/female)	13/4	11/6	0.71 (*χ*^2^ test)
Education level (year)	10.8 ± 4.3	9.9 ± 4.6	0.585
PHES tests			
Final PHES (score)	0.6 ± 1.3	−3.1 ± 2.8	0.028
Number connection test A (seconds)	33.2 ± 9.3	48.0 ± 24.2	0.024
Number connection test B (seconds)	52.7 ± 22.1	88.7 ± 55.2	0.020
Serial dotting test (seconds)	37.5 ± 5.8	41.5 ± 10.5	0.106
Digit symbol test (raw score)	52.1 ± 15.8	41.9 ± 18.4	0.013
Line tracing test (raw score)	113.1 ± 25.8	140.1 ± 31.0	0.001

Abbreviations: HC: healthy control; HCV: hepatitis C virus; PHES: psychometric hepatic encephalopathy score.

**Table 2:** Brain regions with significant ALFF difference between the two study groups.

Regions	Voxels	Brodmann area	MNI coordinates			Peak *T* value
			*x*	*y*	*z*	
Left medial frontal gyrus and bilateral anterior cingulate gyrus	80	10/32	-9	60	9	5.45
Right middle and superior frontal gyrus	63	11/10	30	45	-15	5.58

**Table 3:** Brain regions with significant functional connectivity differences between the two study groups.

Regions	Voxels	Brodmann area	MNI coordinates			Peak *T* value
			*x*	*y*	*z*	
*Decreased connectivity to ROI-1*						
Right middle frontal gyrus	75	8/9	27	30	39	4.43
*Increased connectivity to ROI-2*						
Left supramarginal gyrus and superior temporal gyrus	94	40/22	-60	-54	18	-4.78
Right supramarginal gyrus	60	40	45	-51	36	-4.40

ROI-1: left medial frontal gyrus and bilateral anterior cingulate gyrus; ROI-2: right middle and superior frontal gyrus.

## Data Availability

The data used to support the findings of this study are available from the corresponding author, on reasonable request.

## Abbreviations

HCV: Hepatitis C virus
fMRI: Functional magnetic resonance imaging
ALFF: Amplitude of low-frequency fluctuation
FC: Functional connectivity
PHES: Psychometric hepatic encephalopathy score
FWE: Family-wise error
ROI: Region of interest.
